# Glucose transporter-1 as an independent prognostic marker for cancer: a meta-analysis

**DOI:** 10.18632/oncotarget.18964

**Published:** 2017-07-04

**Authors:** Zheng-Xiao Zhao, Lin-Wei Lu, Jian Qiu, Qiu-Ping Li, Fei Xu, Bao-Jun Liu, Jing-Cheng Dong, Wei-Yi Gong

**Affiliations:** ^1^ Department of Integrative Medicine, Huashan Hospital, Fudan University, Shanghai 200040, P.R. China; ^2^ The Academy of Integrative Medicine of Fudan University, Shanghai 200032, P.R. China

**Keywords:** Glucose transporter-1, GLUT-1, cancer, prognosis, meta-analysis

## Abstract

**Objective:**

Glucose transporter-1 (GLUT-1) as the major glucose transporter present in human cells is found overexpressed in a proportion of human malignancies. This meta-analysis is attempted to assess the prognostic significance of GLUT-1 for survival in various cancers.

**Materials and Methods:**

We conducted an electronic search using the databases PubMed, Embase and Web of Science, from inception to Oct 20th, 2016. Pooled hazard ratios (HRs) and 95% confidence intervals (CIs) were calculated.

**Results:**

Fourty-one studies with a total of 4794 patients were included. High GLUT-1 expression was significantly associated with poorer prognosis [overall survival: HR = 1.833 (95% CI: 1.597–2.069, *P* < 0.0001); disease-free survival: HR = 1.838 (95% CI: 1.264–2.673, *P* < 0.0001); progression-free survival: HR = 2.451 (95% CI: 1.668–3.233, *P* < 0.0001); disease specific survival: HR = 1.96 (95% CI: 1.05–2.871, *P* < 0.0001)].

**Conclusions:**

High GLUT-1 expression may be an independent prognostic marker to predict poor survival in various types of cancers. Further clinical trials with high quality need to be conducted to confirm our conclusion.

## INTRODUCTION

More than 80 years ago, Warburg proposed that one of the most fundamental characteristics of cancer cells is high glucose requirement and increased glucose uptake [1]. Most recently, a number of facilitative glucose transporters have been described to be highly expressed in a wide range of cancer types [2–4]. Glucose transporter (GLUT) family, an expanding family of transmembrane glycol-proteins including GLUT-1 to GLUT-12, is critical for the passive transporting glucose into most cells [5]. Although the metabolic consequences of elevated glucose transporter are not fully understood, the clinical significance of GLUT expression has been illustrated recently.

GLUT-1, a member of glucose transporter family, is originally purified from erythocytes and also found in endothelial cells at the blood-brain barrier, eye, placenta, peripheral nerve and lactating mammary gland [6–7]. It is generally undetectable in normal epithelial cells and benign epithelial tumors, but increased GLUT-1 expression has been observed in a proportion of human malignancies [8–13]. Moreover, experimental studies using *in vitro* models have shown that overexpression of GLUT-1 in cancer cell lines can activate proliferation and survival [14]. In contrast, anti-Glut-1 antibodies result in cell growth inhibition and apoptosis [15]. The mechanisms underlying GLUT-1 regulation in cancer involve in different signaling molecules and pathways, including PI3K/Akt signaling pathway, hypoxia induced factor 1 (HIF-1), Ras, c-Myc and tumor suppressor protein p53 [16]. Numerous reports have suggested that increased GLUT-1 expression has been shown to be associated with poor prognosis in various human cancers [17–22]. However, most studies reporting the implication of GLUT-1 expression are limited in their small sample sizes and discrete outcomes. Therefore, we conduct a systematic review and quantitative meta-analysis to evaluate the prognostic value of GLUT-1 expression as a prognostic marker in human cancers.

## MATERIALS AND METHODS

### Study strategy

The present study was performed according to recent guidelines for meta-analyses and systematic reviews of tumor marker prognostic studies [23–24]. To identify all potential relevant studies, two authors (Wei-yi Gong and Zheng-xiao Zhao) independently searched PubMed, Embase and Web of Science databases to obtain all appropriate articles about GLUT-1 as a prognostic factor for cancer patient survival, without language limitations. The literature search was updated on Oct 20th, 2016. Both Medical Subject Headings and free-text terms, such as “Glucose transporter-1”, “GLUT1”, “Solute carrier family 2A member 1”, “SLC2A1”, “erythrocyte glucose transporter”, “cancer”, “carcinoma”, “tumor”, “prognosis”, “prognostic”, and “survival”, were used to increase the search sensitivity. The bibliographies of the included studies were also searched to identify additional trials.

### Study selection

Two investigators (Wei-yi Gong and Zheng-xiao Zhao) independently screened all eligible studies and extracted the data from included studies. Studies were considered eligible if they fulfilled the following criteria: (1) to deal with human cancer, excepting blood carcinomas; (2) to determine GLUT-1 expression in human tissue using immunohistochemistry (IHC); (3) to examine the relationship between GLUT-1 expression and survival; (4) to provide sufficient data to estimate hazard ratios (HRs) for survival rates and their 95% confidence intervals (CIs); (5) to have been published in English. The studies were excluded if any of the cases occurred: (1) animal studies and single case reports; (2) critical information could not be extracted or calculated from the original article.

### Data extraction

The two investigators (Wei-yi Gong and Zheng-xiao Zhao) extracted data independently. Disagreements were resolved through discussion with a third investigator (Bao-jun Liu). Data on the following characteristics were collected from each article: author, year of publication, country of the population enrolled, number of patients, tumor type, clinical stage of tumor, elevated GLUT-1 expression, cut-off values, overall survival (OS), disease-free survival (DFS), recurrence-free survival (RFS), progression-free survival (PFS), disease-specific survival (DSS), metastasis-free survival (MFS), cancer-specific survival (CSS), and time to progression (TTP).

### Quality assessment of the primary studies

Quality assessment was independently performed by three investigators (Wei-yi Gong, Zheng-xiao Zhao, and Bao-jun Liu) and scored as previously reported [25–26]. Four main methods were evaluated: scientific design, laboratory methodology, generalizability of results, and analysis of the study data. There were four to seven items for each method. Each item was scored as follows: if it was clearly and accurately defined, two points; if it was unclear or incomplete, one point; and if it was not defined or inadequate, zero point. The final scores were expressed as percentages ranging from 0 to 100%, with a higher values reflecting better methodological quality (> 80%).

### Statistical analysis

HRs were extracted using three previously published methods [27–28]. The most accurate method was to obtain hazard ratios (HRs) with their corresponding 95%CIs directly from the published results or to calculate them from the O-E statistic and variance (if available). When both univariate and multivariate Cox regression analyses were reported in the articles, only results of multivariate analysis were selected in the final analysis. If such information was not available, relevant data, such as the number of patients at risk in each group, the number of events, and the log-rank statistics or *p*-values, were used to calculate an approximation of the HRs. However, in some studies, HRs were only displayed in the form of Kaplan-Meier survival curves. Hence we had to evaluate the HRs by extracting survival rates at specified time points from the graphic information as reported previously [28]. In briefly, Engauge Digitizer version 4.1 was used to obtain the necessary points read from the curve. Results were combined as pooled HRs and their 95% CIs. Subgroup analyses were carried out according to the following factors, when appropriate: region, sample size, type of carcinoma, treatment, and quality score.

Meta-regression was generated to explore the possible sources of heterogeneity. They were first estimated using the fixed-effect model to assume heterogeneity. If the heterogeneity was significant (*p* < 0.05 or *I*^2^ > 50%), estimation applying the random-effect model (Mantel-Haenszel) was performed [29].

Sensitivity analysis was applied to test the contribution of some studies to the overall effect and the reliability of the combined results. Sensitivity was assessed in the absence of removing each study. Furthermore, cumulative meta-analyses were conducted to assess the dynamic trends of HRs for OS, DFS and RFS over time.

Publication bias was qualitatively evaluated using funnel plots and quantitatively investigated with Begg's rank correlation test and Egger's regression asymmetry test in the presence of publication bias [30–31]. The statistical analysis was performed using Stata software version 12.0 (Stata, College Station, TX).

## RESULTS

### Data selection and characteristics of eligible studies

**Figure 1:**
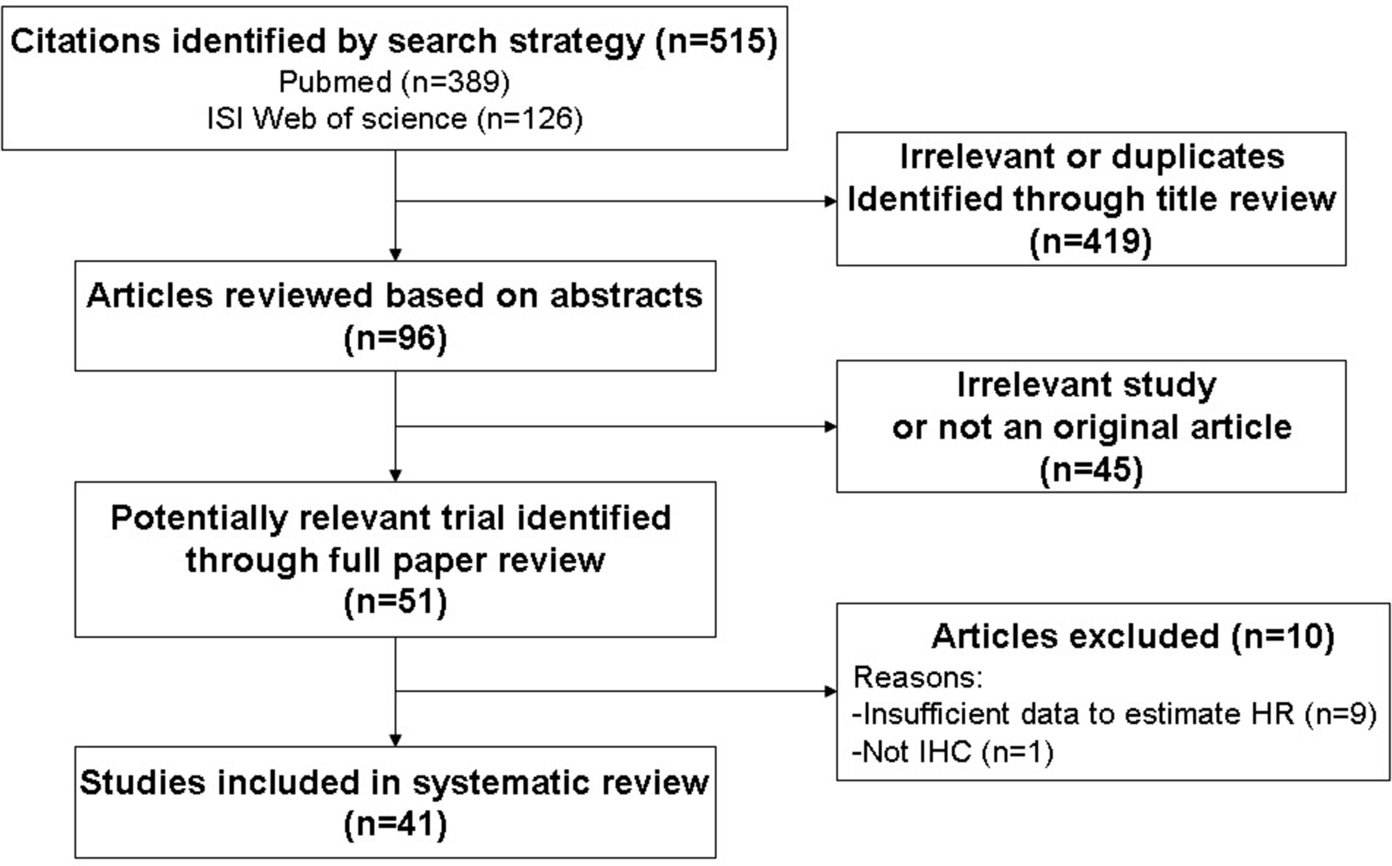
Schemata of the systematic review

We got 515 publications using the literature screening strategy shown in Figure 1. After reviewing titles and abstracts, 464 irrelevant or duplicate studies were excluded. The remaining articles were identified through full paper review and excluded if GLUT-1 expression was not evaluated through IHC or if there were insufficient data to estimate HRs (Supplementary Table 3). Finally, 41 articles were included in the present study.

The detailed characteristics of the included studies are shown in Supplementary Table 1 [17–22, 32–66]. Most of the studies were published in the past ten years (range 2006–2016) nevertheless the exact interval was between 1998 and 2016. We evaluated studies from 14 different countries, including 11 studies from Japan, seven from Korea, six from the United States, five from Germany, and the remaining 12 from ten other countries. A total of 4794 participants were enrolled in these eligible studies, with minimum and maximum sample sizes of 37 and 617 respectively (mean, 116.9 patients).

Twenty-one different types of carcinoma were investigated, most of which were carcinomas of the digestive system (five pancreatic adenocarcinoma, three esophageal squamous cell carcinoma, four each of oral squamous cell carcinoma and colorectal cancer, two each of gastric carcinoma). Other cancer types were also analyzed, including three studies each of ovarian carcinoma and non-small cell lung cancer (NSCLC), two studies on cervix carcinoma and gallbladder carcinomas, and the remaining 12 studies on different types of cancer.

All the studies involve treatment information, and patients underwent surgery were enrolled in 39 studies. Outcome measures were clearly defined in 23 studies, and multivariate analyses were performed in 33 studies (80.5%). OS, RFS, DFS, PFS, MFS and DSS were the primary outcome measures in the included studies. We decided to focus on OS, RFS and DFS. More than half of the included studies (36/41, 87.8%) scored a quality of ≥ 80%.

A total of 51 HRs were from 41 studies, including 30 for OS; seven for DFS; four for RFS; three for DSS; two for MFS and PFS; one for TTP, CSS, EFS. Among these, 26 HRs were directly acquired and 5 were estimated from the total number of events and the log-rank statistics or *p*-values. The remaining 20 were estimated from Kaplan-Meier survival curves. High or positive GLUT-1 expression was identified as an indicator of poor OS (29/30, 96.7%), DFS (6/7, 85.7%), RFS (3/4, 75%), DSS (3/3, 100%).

### The prognostic significance of high GLUT-1 expression in OS in human cancer

**Figure 2:**
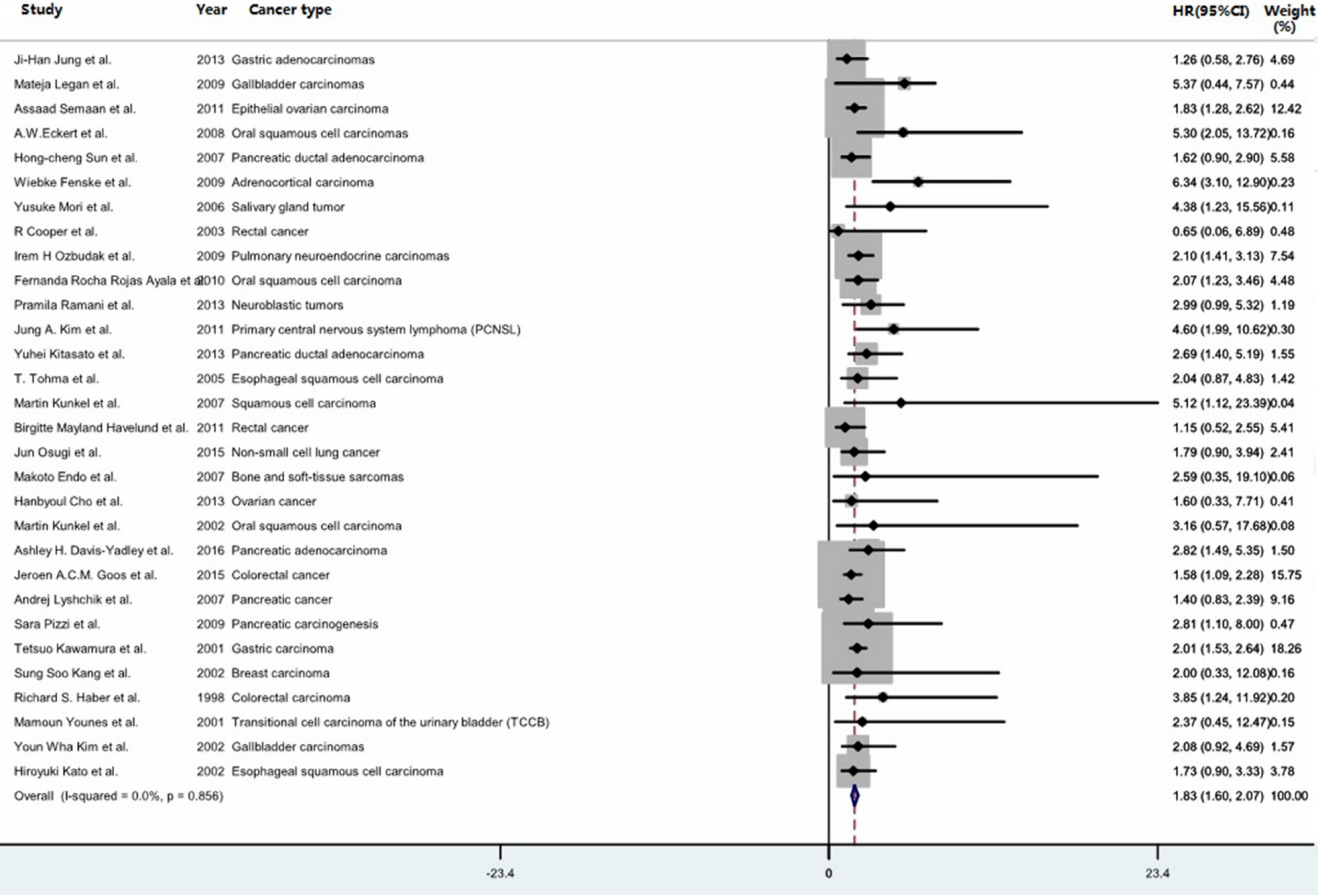
Forest plot for the meta-analysis of the association between GLUT-1 expression and overall survival in various cancer types The segments represent the 95% confidence intervals (CIs) of each study. The diamond represents the overall effect size, and the diamond's width represents the overall 95% CI.

**Figure 3:**
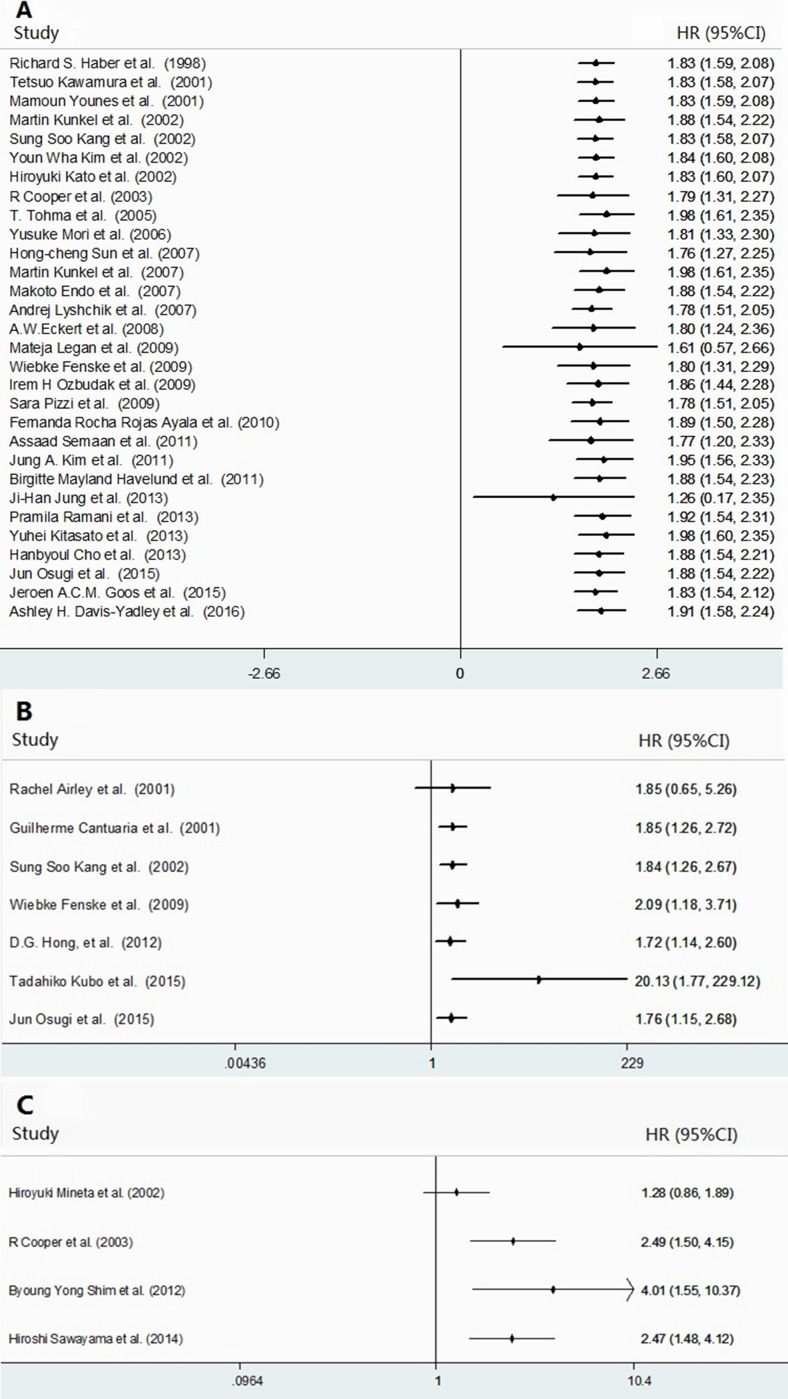
Forest plots for the accumulative meta-analyses of the association between GLUT-1 expression and cancer survival The following cancer survival measures were analyzed: OS (**A**), DFS (**B**), and RFS (**C**). The segments represent the 95% confidence interval (CIs) of each study. The diamond represents the overall effect size, and the diamond's width represents the overall 95% CI.

The correlation between GLUT-1 expression and OS was performed in aggregative 30 literatures enrolling 3528 patients with various cancer types [18–22, 32, 37–40, 42, 44–54, 57–58, 60–63, 65–66]. The overall analysis showed that high GLUT-1 expression was associated with poor OS in cancer (HR = 1.833, 95% CI: 1.597–2.069; *P* < 0.0001) with no significant heterogeneity (*I*^2^ = 0%) (Table 1 and Figure 2). The cumulative meta-analysis showed that HRs was rater stable over time (Figure 3A). Such results demonstrated that high GLUT-1 expression was an independent predictor for poor OS in multiple cancers.

**Table 1:** Results of subgroup analysis of the association between GLUT-1 expression and OS of multiple cancers

Subgroup analysis	No. of studies	No. of patients	Pooled HR	*p* value	Meta regression (*p*-value)	Heterogeneity	
						*I*^2^	*p*-value (χ^2^)
**Overall survival**	30	3528	1.833 [1.597–2.069]	< 0.0001		0%	0.856
**Region**					< 0.0001		
Asian countries	14	1725	1.793 [1.458–2.129]	< 0.0001		0%	0.954
Western countries	16	1803	1.872 [1.54–2.204]	< 0.0001		1.25%	0.438
**Sample size**					< 0.0001		
< 150	19	1207	1.801 [1.395–2.207]	< 0.0001		0%	0.698
≥ 150	11	2321	1.849 [1.559–2.14]	< 0.0001		0%	0.766
**Type of cancer**					< 0.0001		
Gastric cancer	2	810	1.858 [1.365–2.351]	< 0.0001		30.70%	0.23
Urinary carcinoma	2	96	4.589 [1.523–7.655]	0.003		0%	0.4
Ovarian carcinoma	2	277	1.823 [1.163–2.482]	< 0.0001		0%	0.904
Oral squamous cell carcinomas	4	342	2.224 [1.141–3.306]	< 0.0001		0%	0.694
Pancreatic adenocarcinoma	5	296	1.729 [1.177–2.282]	< 0.0001		0%	0.506
Colorectal cancer	4	591	1.473 [0.968–1.979]	< 0.0001		0%	0.683
Lung cancer	2	290	2.026 [1.278–2.775]	< 0.0002		0%	0.732
Gallbladder carcinomas	2	127	3.363 [0.218–6.508]	0.036		60.90%	0.11
Esophageal squamous cell carcinoma	2	158	1.815 [0.779–2.85]	0.001		0%	0.794
Treatment					< 0.0001		
Surgery without preoperative treatment	22		1.911 [1.653–2.168]	< 0.0001		0%	0.872
Surgery with preoperative treatment	8		1.417 [0.821–2.013]	< 0.0001		0%	0.672
**Quality score (%)**					< 0.0001		
< 83.0	14		1.916 [1.611–2.22]	< 0.0001		0%	0.538
≥ 83.0	16		1.709 [1.336–2.083]	< 0.0001		0%	0.902

OS: overall survival; HR: hazard ratio.

Subgroup analysis indicated high GLUT-1 expression was significantly associated with poor OS in gastric cancer (HR = 1.858, 95%CI: 1.365–2.351; *P* < 0.0001), urinary carcinoma (HR = 4.589, 95% CI: 1.523–7.655; *P* = 0.003), ovarian carcinoma (HR = 1.823, 95%CI: 1.163–2.482; *P* < 0.0001); oral squamous cell carcinomas (HR = 2.224, 95% CI: 1.141–3.306; *P* < 0.0001); pancreatic adenocarcinoma (HR = 1.729, 95% CI: 1.177–2.282; *P* < 0.0001); colorectal cancer (HR = 1.473, 95% CI: 0.968–1.979; *P* < 0.0001); lung cancer (HR = 2.026, 95% CI: 1.278–2.775; *P* < 0.0002), gallbladder carcinoma (HR = 3.363, 95% CI: 0.218–6.508; *P* = 0.036), esophageal squamous cell carcinoma (HR = 1.815, 95%CI: 0.779–2.85; *P* = 0.001). These results were partly consistent with the previous study reported by Xiu Chen et al, which found the correlation of GLUT-1 up-regulation and negative OS in pancreatic and gastric cancer but not in colorectal cancer [67]. Unfortunately, we could not gather information from the other 12 cancer types because only a single study was involved in each subgroup. The combined results of HR were significantly higher for studies those of patients undergoing surgery without preoperative treatment (no preoperative vs. preoperative therapy: HR = 1.911, 95%CI: 1.653–2.168; *P* < 0.0001). The pooled HRs were greater in studies with poor quality than in studies with better quality (HR = 1.916, 95% CI: 1.611–2.22; *P* < 0.0001). The results of studies with larger sample sizes were similar to those with smaller (≥ 150 vs. <150: HR 1.849, 95% CI: 1.559–2.14; *P* < 0.0001). The pooled HRs for both Western and Asian patients were also alike (Western vs. Asian: HR = 1.872, 95% CI: 1.54–2.204; *P* < 0.0001).

Meta-regression analysis revealed that region, sample size, type of cancer, treatment and quality score might contribute significantly to heterogeneity (*P* < 0.0001).

### The prognostic significance of high GLUT-1 expression in DFS, RFS, PFS, DSS and MFS in human cancer

Overall analyses of the associations between high GLUT-1 expression and DFS, RFS, PFS, DSS and MFS were presented in Tables 2 and 3 [17–18, 20, 33, 35–36, 38–39, 41, 43, 49, 55–56, 59, 61, 64]. The elevated expression of GLUT-1 expression was found to be significantly correlated with poor DFS (Supplementary Figure 1), PFS and DSS, but neither MFS nor RFS. Statistically significant heterogeneity was observed across the studies for DFS, RFS, PFS, DSS and MFS (Tables 2 and 3). Cumulative meta-analysis of DFS and RFS revealed that all the HRs were quite stable (Figure 3B and 3C).

**Table 2:** Results of subgroup analysis of the association between GLUT-1 expression and DFS and RFS of multiple cancers

Subgroup analysis	No. of studies	No. of patients	Pooled HR	*p* value	Meta regression (*p*-value)	Heterogeneity	
						*I*^2^	*p*-value (χ^2^)
**Disease-free survival**	7		1.838 [1.264–2.673]	0.001		8.5%	0.364
**Region**					< 0.0001		
Asian countries	3	275	1.433 [0.529–2.337]	0.002		0%	0.966
Western countries	4	688	1.714 [0.632–2.796]	0.002		0%	0.683
**Sample size**					< 0.0001		
< 100	2	95	1.042 [0.37–2.93]	0.939		0%	0.89
≥ 100	5	868	1.69 [0.902–2.477]	< 0.0001		0%	0.883
**Type of cancer**					< 0.0001		
Gynecologic oncology	3	208	1.729 [0.821–3.64]	0.149		0%	0.38
NSCLC	2	469	1.441 [0.491–2.391]	0.003		0%	0.747
**Quality score**					< 0.0001		
< 83	3	281	1.563 [0.468–2.659]	0.005		0%	0.598
≥ 83	4	682	1.539 [0.642–2.435]	0.001		0%	0.875
**Recurrence-free survival**	4	391	1.63 [0.515–5.158]	0.405		83.1%	< 0.0001
**Publication year**					< 0.0001		
< 2010	2	249	0.486 [0.169–0.803]	0.003		0%	0.96
≥ 2010	2	142	2.181 [0.929–3.434]	0.001		0%	0.397
**Sample size**					< 0.0001		
< 100	2	142	0.486 [0.169–0.803]	0.003		0%	0.96
≥ 100	2	249	2.181 [0.929–3.434]	0.001		0%	0.397
**Type of cancer**							
Rectal cancer	2	147	4.107 [1.609–10.482]	0.003		0%	0.769
**Quality score**					< 0.0001		
< 83	2	249	2.181 [0.929–3.434]	0.001		0%	0.397
≥ 83	2	142	0.486 [0.169–0.803]	0.003		0%	0.96

DFS: disease-free survival; RFS: recurrence-free survival; HR: hazard ratio.

**Table 3:** Results of the meta-analysis of the association between GLUT-1 expression and PFS, DSS and MFS of multiple cancers

Meta-analysis	No. of studies	Cancer type	No. of patients	Pooled HR	*p* value	Heterogeneity	
						*I*^2^	*p*-value (χ^2^)
**Progression-free survival**	2	Locally advanced cervical squamous cell carcinoma (LACSCC), epithelial ovarian carcinoma	345	2.451 [1.668–3.233]	< 0.0001	0%	0.34
**Disease specific survival**	3	Oral squamous cell carcinomas (OSCCs), NSCLC	541	1.96 [1.05–2.871]	< 0.0001	0%	0.872
**Metastasis-free survival**	2	Cervix carcinoma, rectal cancer	97	0.491 [0.128–1.891]	0.301	0%	0.609

PFS: progression-free survival; DSS: disease specific survival; HR: hazard ratio.

The predictive role of GLUT-1 for DFS was significant for all subgroups except for studies of smaller size and patients with gynecologic system malignancy (Table 2). On the other hand, the correlation between GLUT-1 and RFS was significant in all subgroups (Table 2). We did not carry out subgroup analysis for PFS, DSS and MFS, due to the limited number of studies (two or three) on these outcomes.

Meta-regression analysis was performed for DFS, indicating that region, sample size, type of cancer and quality score were significantly responsible for the bias among studies. Also, meta-regression analysis was performed for RFS, demonstrating that the bias came from the publication year, sample size and quality score.

### Analysis of sensitivity and publication bias

Chemoradiotherapy rather than surgery was given to patients in the study conducted by Jung A. Kim et al. (2011) [44]. Thus, we excluded this studies when reported a sensitivity analysis of OS, and our result was proven to be stable, but the exclusion of this report did not significantly alter the results (HR = 1.833, 95% CI: 1.597–2.069; *P* < 0.0001 and HR = 1.825, 95% CI: 1.588–2.061; *P* < 0.0001, respectively) (Supplementary Figure 2A). Similarly, the sensitivity analyses showed that the pooled HRs of DFS were reliable (Supplementary Figure 2B). In the sensitivity analysis of RFS, the report by Hiroyuki Mineta et al. (2002) affected the whole stability. We excluded this study and did meta-analysis again, the heterogeneity dropped from 59.1% to 0% (HR = 0.588, 95%CI: 0.281–0.895; *P* < 0.0001 and HR = 2.181, 95%CI: 0.929–3.434; *P* = 0.001, respectively) (Supplementary Figure 2C).

No significant publication bias was detected in for the meta-analysis of the association between GLUT-1 and OS, as indicated by Egger's test (*P* = 0.058), Begg's test (*P* = 0.101) and relatively symmetrical appearance of the funnel plot (Supplementary Table 2 and Supplementary Figure 3). Moreover, no evidence of publication bias was observed in the subgroups (Supplementary Table 2). Consistently, there was no evidence of publication bias for either DFS or RFS (DFS, *P* = 0.487 via Egger's test and *P* = 0.368 via Begg's test; RFS, *P* = 0.634 via Egger's test and *P* = 1.000 via Begg's test) (Supplementary Table 2).

## DISCUSSION

This study aimed to disclose the prognostic value of GLUT-1 expression in cancer survival by examining the correlation between GLUT-1 and various survival measures. We found a reciprocal relationship between elevated GLUT-1 expression and OS, DFS, PFS and DSS, but neither MFS nor RFS. These results suggested that GLUT-1 might be an independent prognostic marker for diverse types of cancers.

We performed subgroup analysis and the results revealed that patients with high GLUT-1 expression were more likely to have poor OS in gastric cancer, urinary carcinoma, ovarian carcinoma, oral squamous cell carcinoma, pancreatic adenocarcinoma, colorectal cancer, lung cancer, gallbladder carcinoma, esophageal squamous cell carcinoma. Nevertheless, these positive results were only based on a relatively small number of studies (two to five), and for the other types of cancer, only one study was included. In a previous meta-analysis that analyzed eight independent studies comprising data from 921 patients [68], it was observed that the GLUT-1 overexpression was in connection with worse OS in oral squamous cell carcinoma (OSCC) (HR = 1.88, 95% CI: 1.51–2.33, *P* < 0.001), which was similar to our result (HR = 2.224, 95% CI: 1.141–3.306; *P* < 0.0001).

In the current study, subgroup analyses proved that patients with preoperative treatment had a significantly shorter OS than those without preoperative treatment. This finding might imply the intervening role of preoperative treatment in the association between GLUT-1 and cancer survival (owing to its survival disadvantages). Given this, we should take the preoperative treatment into account when applying GLUT-1 as a predictor to assess the OS in cancer patients. Further investigations should be conducted to verify our results, because the publication biases could be derived from the limited quantity of studies with preoperative treatment.

In addition, our results demonstrated that high GLUT-1 expression was more closely related to poor DFS in Western patients than in Asian populations, and similar significant predictive value was also seen in OS. In the light of higher quality paper, the results indicated that high GLUT-1 expression was neither associated with OS, nor with DFS. Moreover, our analysis of HRs were larger in large studies than in small studies. Thus, we might have disappreciated the prognostic significance of GLUT-1 in cancer survival, as a consequence of the inequality in the contribution of results from low-quality or relatively small studies. Similarly, the results revealed that the associations of GLUT-1 with PFS, DSS and MFS were based on only two or three studies. Therefore, the conclusions drawn from small sample size were not accurate and objective at present.

Meta-regression and sensitivity analyses did not affect the significant association of GLUT-1 with worse survival or reveal any significant sources of heterogeneity. Cumulative meta-analyses exhibited an insignificant trend towards increased hazard for OS, DFS or RFS over time. As a result, the analyses showed no significant publication bias, despite one or two articles reporting results that departed from the steady trend.

However, several limitations included in our study cannot be overlooked. Firstly, this study analyzed the prognostic values of GLUT-1 in diverse cancers, rather than a single specific type. This might generate remarkable bias due to different baseline characteristics of various cancer types. Secondly, cancer staging and the criteria for calculating GLUT-1 cut-off values were inconsistent across studies, and the definitions of outcome measures were not available in all reports. In the present study, we defined GLUT-1 expression greater than the corresponding cut-off values as high or positive, while other status as low or negative. This simple approach for classification might have introduced obvious heterogeneity. Thirdly, treatments other than surgery were involved in the enrolled patients, which may increase the baseline heterogeneity. Fourthly, there was inevitable bias in our analysis of the association between high GLUT-1 expression and OS, due to the lack of sufficient number of studies. Furthermore, studies reported in non-English language papers, unpublished studies, and conference abstracts were not included. Therefore, our results might overestimate the prognostic value of GLUT-1 in the prognosis of patients with cancer, owing to the incomplete data collection. Fifthly, we obtained five estimates by calculation and 20 by survival curve reconstruction rather than directly acquired data from the primary studies, leading to the inevitably considerable bias.

In conclusion, to the best of our knowledge, this was the first study on the prognostic value of GLUT-1 in diverse cancer types. Our meta-analysis revealed that GLUT-1 might be a significant predictor for OS, DFS, PFS and DSS in multiple types of cancer. Thus, it might serve as a novel effective biomarker for early diagnosis or prognostic prediction. However, the exact predicting role should be further confirmed in high-quality prospective clinical trails.

## SUPPLEMENTARY MATERIALS FIGURES AND TABLES




