# Supplementary material for: Glucose transporter-1 as an independent prognostic marker for cancer: a meta-analysis
Source: Oncotarget. 2017 Jul 4;9(2):2728–38. doi: 10.18632/oncotarget.18964 (PMC5788674; doi:10.18632/oncotarget.18964)
Supplement: Supplementary file 1 [file oncotarget-09-2728-s001.pdf]

Glucose transporter-1 as an independent prognostic marker for cancer: a meta-analysis

SUPPLEMENTARY MATERIALS

Supplementary Table 1: Detailed characteristics of studies included in the meta-analysis. See Supplementary_Table_1

Supplementary Table 2: Publication bias regarding the analysis of the association between GLUT-1 expression and OS, DFS and RFS

Outcome measures	Egger's test		Begg's or Begg-Mazumdar test		Presence of publication bias
	t	*p* value	z (continuity corrected)	*p* value (continuity corrected)	
**OS**	1.97	0.058	1.64	0.101	Non-significant
**Region: Asian countries**	0.63	0.538	1.31	0.189	Non-significant
**Region: Western countries**	1.95	0.071	1.04	0.300	Non-significant
**Sample size < 150**	1.41	0.176	0.98	0.327	Non-significant
**Sample size ≥ 150**	1.04	0.327	0.62	0.533	Non-significant
**Surgery without preoperative treatment**	1.94	0.066	1.86	0.063	Non-significant
**Surgery with preoperative treatment**	0.78	0.464	−0.12	1.000	Non-significant
**Quality score < 83.0**	1.84	0.091	1.53	0.125	Non-significant
**Quality score ≥ 83.0**	1.18	0.256	0.41	0.685	Non-significant
**DFS**	0.75	0.487	0.9	0.368	Non-significant
**RFS**	0.56	0.634	−0.34	1.000	Non-significant

OS: overall survival; DFS: disease-free survival; RFS: recurrence-free survival.

Supplementary Table 3: Summary of the excluded studies and the reasons for exclusion

Articles	Reasons for exclusion
1. Marcelo Gadelba Vasconcelos, Rodrigo Gadelba Vasconcelos, Denise Helen Imaculada Pereira de Oliveira, et al. Distribution of Hypoxia-Inducible Factor-1α and Glucose Transporter-1 in Human Tongue Cancers. J Oral Maxillofac Surg. 73:1753–1760.	No sufficient data to calculate or approximate HR estimates
2. Ji-Youn Sung, Gou Young Kim, Sung-Jig Lim, et al. Expression of the GLUT1 glucose transporter and p53 in carcinomas of the pancreatobiliary tract. Pathology–Research and Practice. 206:24–29.	No sufficient data to calculate or approximate HR estimates
3. Xuan Canh Nguyen, Won Woo Lee, Jin-Haeng Chung, et al. FDG uptake, glucose transporter type 1, and Ki-67 expressions in non-small-cell lung cancer: Correlations and prognostic values. European Journal of Radiology. 62:214–219.	No sufficient data to calculate or approximate HR estimates
4. Jieun Lee, Jung Oh Kim, Chan Kwon Jung, et al. Metabolic Activity on [18F]-Fluorodeoxyglucose- Positron Emission Tomography and Glucose Transporter-1 Expression Might Predict Clinical Outcomes in Patients With Limited Disease Small-Cell Lung Cancer Who Receive Concurrent Chemoradiation. Clinical Lung Cancer. 15:e13–21.	No sufficient data to calculate or approximate HR estimates
5. Bo Wook Kim, Hanbyoul Cho, Joon-Yong Chung, et al. Prognostic assessment of hypoxia and metabolic markers in cervical cancer using automated digital image analysis of immunohistochemistry. Journal of Translational Medicine. 11:185.	No sufficient data to calculate or approximate HR estimates
6. Susumu Saigusa, Yuji Toiyama, Koji Tanaka, et al. Prognostic significance of glucose transporter-1 (GLUT1) gene expression in rectal cancer after preoperative chemoradiotherapy. Surg Today. 42:460–469.	No sufficient data to calculate or approximate HR estimates
7. Andrzej Wincewicz, Mariola Sulkowska, Mariusz Koda, et al. Significant Coexpression of GLUT-1, Bcl-xL, and Bax in Colorectal Cancer. Ann N Y Acad Sci. 1095:53–61.	No sufficient data to calculate or approximate HR estimates
8. Katarzyna Starska, Ewa Forma, Pawel Jozwiak, et al. Gene and protein expression of glucose transporter 1 and glucose transporter 3 in human laryngeal cancer – the relationship with regulatory hypoxia-inducible factor-1α expression, tumor invasiveness, and patient prognosis.	GLUT-1 expression (mRNA) with methods other than IHC
9. Celine Pinheiro, Barbara Sousa, Andre Albergaria, et al. GLUT1 and CAIX expression profiles in breast cancer correlate with adverse prognostic factors and MCT1 overexpression. Histol Histopathol 26:1279–1286.	No sufficient data to calculate or approximate HR estimates
10. Mamoun Younes, Richard W. Brown, Mark Stephenson, et al. Overexpression of Glut1 and Glut3 in Stage I Nonsmall Cell Lung Carcinoma Is Associated with Poor Survival. Cancer. 80:1046–1051.	No sufficient data to calculate or approximate HR estimates

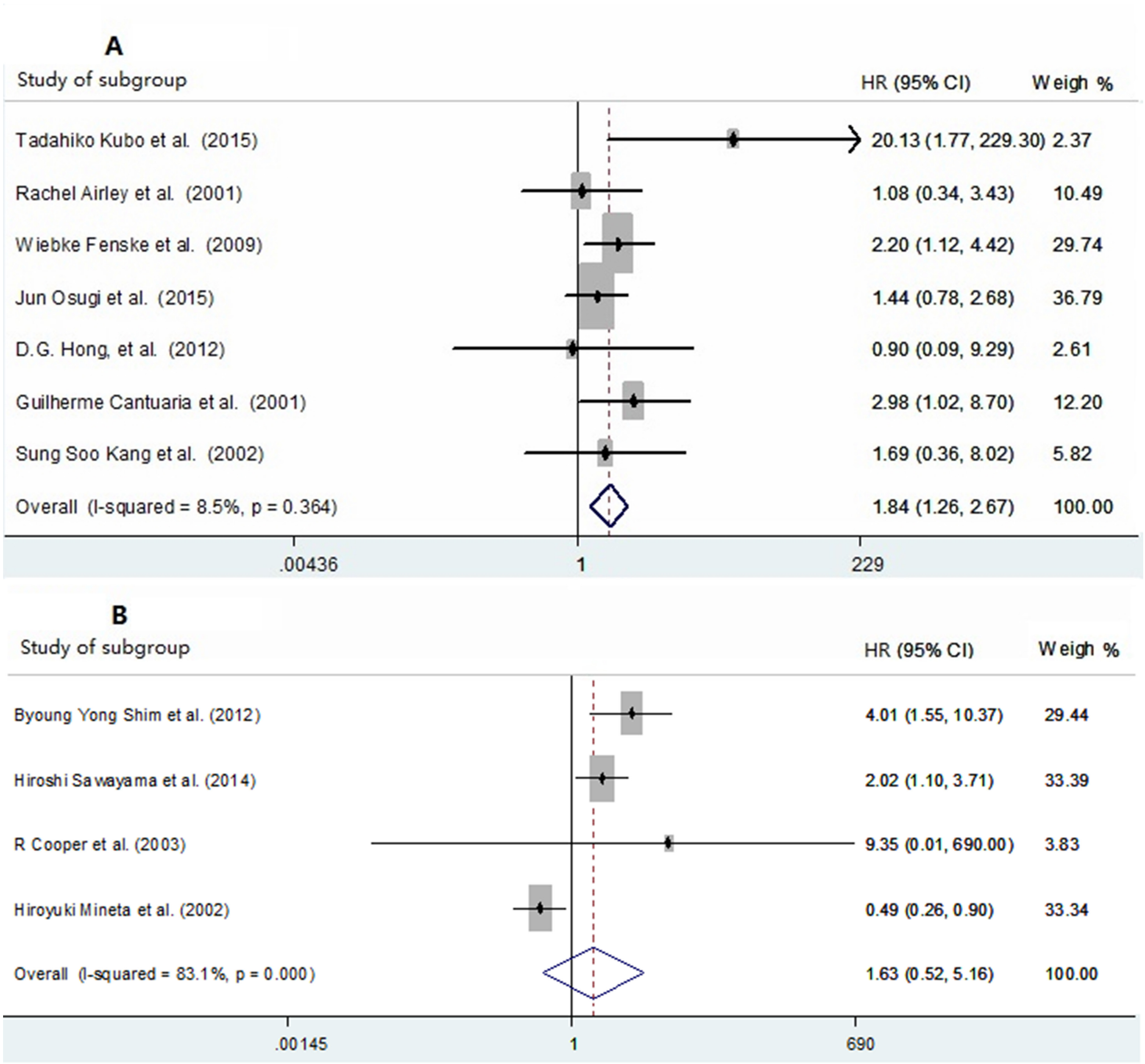

Supplementary Figure 1: Forest plots for the meta-analysis of the association between GLUT-1 expression and cancer survival. The following cancer survival measures were analyzed: DFS (**A**) and RFS (**B**). The segments represent the 95% confidence intervals (CIs) of each study. The diamond represents the overall effect size, and the diamond's width represents the overall 95% CI.

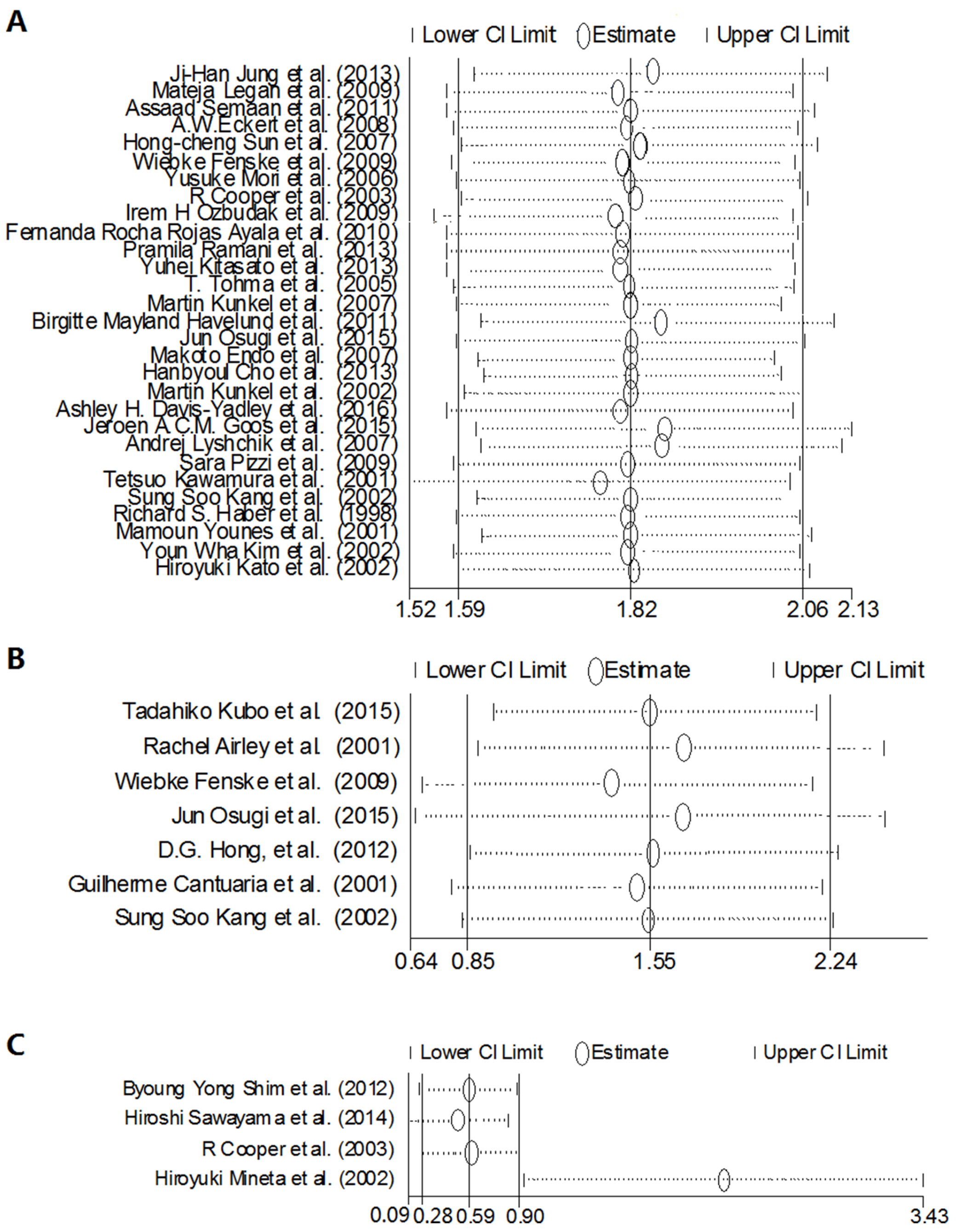

Supplementary Figure 2: Sensitivity analysis. Pooled relative risk and 95% confidence intervals (CIs) by omitting each study. (**A**) For OS group; (**B**) For DFS group; (**C**) For RFS group.

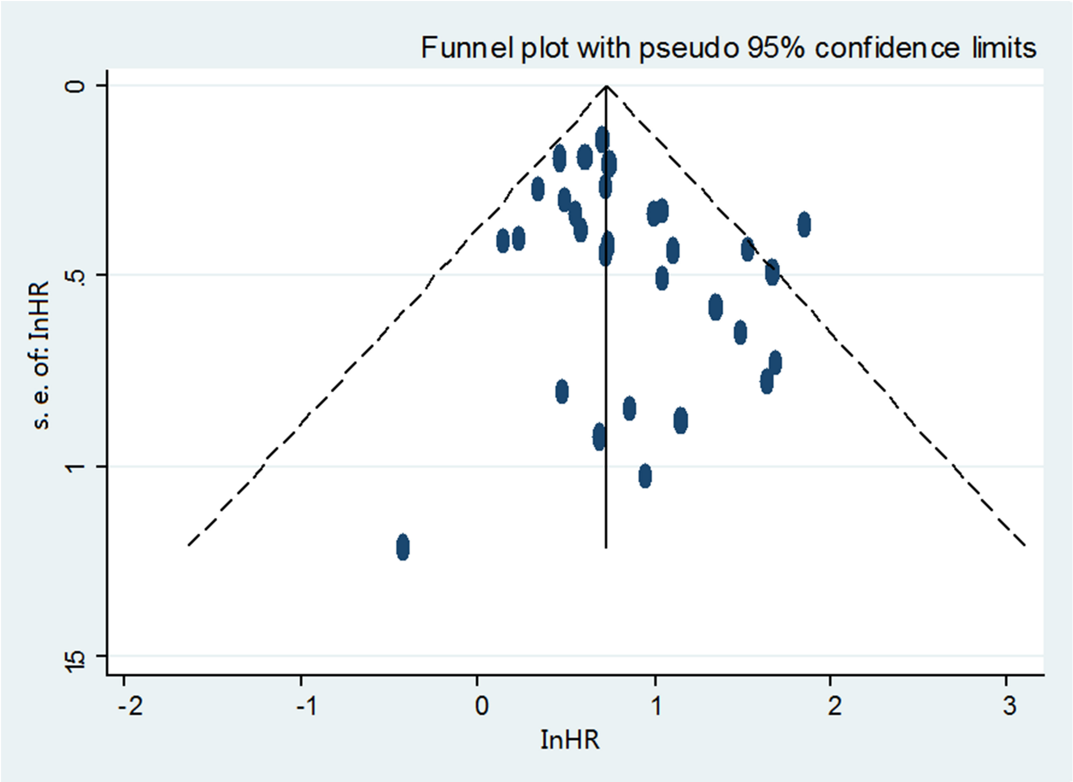

Supplementary Figure 3: Funnel plot for the assessment of publication bias in our analysis of the correlation between GLUT-1 expression and overall survival in various cancer types.
