# Supplementary material for: Glucose transporter-1 as an independent prognostic marker for cancer: a meta-analysis
Source: Oncotarget. 2017 Jul 4;9(2):2728–38. doi: 10.18632/oncotarget.18964 (PMC5788674; doi:10.18632/oncotarget.18964)
Supplement: Supplementary file 2 [file oncotarget-09-2728-s002.doc]

**Supplementary Table 1: Detailed characteristics of studies included in the meta-analysis**

| **Study** | **Year** | **Region** | **Tumor type** | **Sample size (n)** | **Clinical stage of tumor** | **Cut-off value** | **Elevated GLUT-1 (%)** | **Treatment** | **Outcome measure** | **Definition of outcome measures** | **Survival analysis** | **Quality score**  **(%)** |
| --- | --- | --- | --- | --- | --- | --- | --- | --- | --- | --- | --- | --- |
| Ji-Han Jung et al. | 2013 | Korea | Gastric adenocarcinomas | 193 | I-IV (7th AJCC classification) | Low vs high: staining index: 0-1 vs 2-4 | 43.00% | Surgery, no preoperative treatment | OS | OS: from the date of initial surgery to the date of death | Univariate and multivariate analysis | 84.30% |
| Xin-Qiong Huang et al. | 2014 | China | Locally advanced cervical squamous cell carcinoma (LACSCC) | 132 | FIGO stages IB-IVA | Low vs high: a staining score: 0-4 vs 5-12 | 61.40% | Primary radiation therapy | PFS | PFS: period from the end of therapy to the date of the first documented evidence of recurrence or metastatic disease | Univariate and multivariate analysis | 81.40% |
| Alexander W Eckert et al. | 2011 | Germany | Oral squamous cell carcinomas (OSCCs) | 79 | TNM I-IV (AJCC TNM classification system) | Low vs high: immunoreactive score (IRS): 0-4 vs 6-12 | 59.00% | Surgery, or surgery with radiotherapy, or surgery with radiotheapy and chemotherapy, or radiotherapy only, or radiotherapy with chemotherapy | DSS | NA | Univariate and multivariate analysis | 82.10% |
| Sigve Andersen et al. | 2010 | Norway | Non-small cell lung cancer | 335 | I-IIIA (TNM classification) | Low vs high: a staining score: ≤1 vs ＞1 | 77.00% | Surgery,no preoperative treatment | DSS | DSS: from the time of surgery to the time of lung cancer death | Univariate and multivariate analysis | 86.30% |
| Tadahiko Kubo et al. | 2015 | Japan | Osteosarcoma | 37 | AJCC | Positive vs negative: staining in 10% of cells | 32.40% | Surgery with conventional chemotherapy | DFS | NA | Univariate and multivariate analysis | 80.70% |
| Mateja Legan et al. | 2009 | Slovenia | Gallbladder carcinomas | 56 | Grade I-III | Positive vs negative: staining in 50% of cells | 51.80% | Surgery | OS | NA | Univariate and multivariate analysis | 76.20% |
| Assaad Semaan et al. | 2011 | USA | Epithelial ovarian carcinoma | 213 | FIGO stages I-IV | Low vs high: intensity of 1-3 and ＜50% stained cells vs intensity of 1-3 and ＞50% stained cells | 23.50% | Surgery,no preoperative treatment | OS, PFS | PFS: the time interval from the date of primary surgery to the date of disease progression and/or recurrence. OS: the time from the date of primary surgery to the date of death or censored at the date of last contact in months | Univariate and multivariate analysis | 79.10% |
| A.W.Eckert et al. | 2008 | Germany | Oral squamous cell carcinomas | 42 | Stage I-IV | Negative or weak vs moderate or strong: 0-4 vs 6-12 | 23.80% | Surgery with or without radiotherapy | OS | NA | Multivariate analysis | 77.30% |
| Hong-cheng Sun et al. | 2007 | China | Pancreatic ductal adenocarcinoma | 58 | UICC TNM classification I-IV | Low reactivity group vs high reactivity group: - +/++ +++ | 67.20% | Surgery,no preoperative treatment | OS | OS: from the day of surgery until death of the patient | Univariate and multivariate analysis | 76.60% |
| Byoung Yong Shim et al. | 2012 | Korea | Rectal cancer | 104 | Stage I-III | Low vs high: staining index: 0-1 vs 2-3 | 21.20% | Neoadjuvant chemoradiotherapy and surgery | RFS | NA | Univariate and multivariate analysis | 81.70% |
| Rachel Airley et al. | 2001 | New Zealand | Cervix carcinoma | 54 | Stage I-IV | Absent vs present: staining index: 0 vs 1-3 | NA | Surgery | DFS, MFS | NA | Bivariate analysis | 77.80% |
| Wiebke Fenske et al. | 2009 | Germany | Adrenocortical carcinoma | 186 | WHO stage I-IV | Low vs high: staining index: 0-1 vs 2 | 8.60% | Surgery | OS, DFS | NA | Univariate and multivariate analysis | 80.80% |
| Yusuke Mori et al. | 2006 | Japan | Salivary gland tumor | 97 | NA | Low vs high: GLUT1 labeling index: <15% vs≥15% | 69.20% | Surgery | OS | NA | Univariate and multivariate analysis | 82.30% |
| R Cooper et al. | 2003 | Turkey | Rectal cancer | 43 | Duke's stage B/C | Negative vs positive: 0 vs 1-3 | 70.00% | Surgery with or without preoperative radiotherapy/chemotherapy and/or postoperative radiotherapy/chemotherapy | OS, MFS, RFS | NA | Bivariate analysis | 84.50% |
| Irem H Ozbudak et al. | 2009 | USA | Pulmonary neuroendocrine carcinomas | 156 | stage I-IV | Negative vs positive: 0 vs 1-3 | 46.60% | Surgery | OS | NA | Univariate and multivariate analysis | 81.70% |
| Fernanda Rocha Rojas Ayala et al. | 2010 | Brazil | Oral squamous cell carcinoma | 142 | stage I-IV | Negative vs positive: <10% vs ≥10% | 95.10% | Surgery | OS | NA | Univariate and multivariate analysis | 82.40% |
| Pramila Ramani et al. | 2013 | UK | Neuroblastic tumors | 96 | INSS stage I-IV | Absent vs present: staining index: <3% vs ≥3% | 46.00% | Surgery | OS, EFS | OS: from the time of diagnosis to death. EFS: from the time of diagnosis until the time of the first occurrence of relapse, progression, secondary malignancy or death, if none of the former had occurred. | Univariate and multivariate analysis | 83.90% |
| Hiroshi Sawayama et al. | 2014 | Japan | Esophageal squamous cell carcinoma | 145 | TNM 7th edition IA-IIIA | Negative vs positive: staining <50% vs ≥50% | 28.20% | Surgery without preoperative treatment | RFS, CSS | NA | Multivariate analysis | 82.60% |
| Jung A. Kim et al. | 2011 | Korea | Primary central nervous system lymphoma (PCNSL) | 51 | WHO classification | Negative vs positive: <20% vs ≥20% | 33.30% | Treated with high-dose MTX-based chemotherapy and radiotherapy | OS, TTP | OS: from the date of diagnosis to the date of death or last follow-up. TTP: from the date at which treatment began until the date when disease progression or disease relapse was recognized. | Kaplan-Meier method and compare by log-rank test | 83.10% |
| Yuhei Kitasato et al. | 2013 | Japan | Pancreatic ductal adenocarcinoma | 41 | UICC stage I-IV | Low vs high: staining 0-1+ vs 2+-3+ | 48.80% | Surgery | OS | NA | Kaplan-Meier method | 83.00% |
| T. Tohma et al. | 2005 | Japan | Esophageal squamous cell carcinoma | 63 | TNM classification | Low vs high: staining <30% vs >30% | 77.80% | Surgery | OS | NA | Kaplan-Meier method | 81.40% |
| Martin Kunkel et al. | 2007 | Germany | Squamous cell carcinoma | 40 | AJCC/UICC stage I-IV | Low vs high: labeling index (LI) <median value vs >median value | NA | Preoperative radiotherapy followed by surgical resection | OS | NA | Kaplan-Meier method | 81.70% |
| Birgitte Mayland Havelund et al. | 2011 | Denmark | Rectal cancer | 86 | T3 or T4 stage | Low vs high: IRS <3 vs IRS≥3 | NA | Preoperative radiotherapy and concomitant chemotherapy | OS | OS: the time from operation until death from any cause | Univariate analysis | 82.90% |
| Jun Osugi et al. | 2015 | Japan | Non-small cell lung cancer | 134 | Stage I-III | Negative vs positive: <50% vs ≥50% | 56.00% | Surgery without preoperative or adjuvant treatment | OS, DFS | OS: the time from surgery to last follow-up or time of NSCLC-specific death. DFS: the time from surgery to the time of first evidence of radiographic metastatic disease. | Univariate and multivariate analysis | 84.30% |
| Makoto Endo et al. | 2007 | Japan | Bone and soft-tissue sarcomas | 67 | WHO classification system: IA-IVB | Negative vs positive: <10% vs ≥10% | 96.00% | Surgery with adjuvant chemotherapy and/or radiotherapy | OS | OS: the time from diagnosis to death from any cause. | Univariate and multivariate analysis | 84.10% |
| Hanbyoul Cho et al. | 2013 | Korea | Ovarian cancer | 64 | FIGO staging system | Negative vs positive: IHC score<3.85 vs ≥3.85 | 78.10% | Surgery | OS | NA | Kaplan-Meier method | 85.40% |
| Martin Kunkel et al. | 2002 | Germany | Oral squamous cell carcinoma | 118 | UICC staging category I-IV | Low vs high: LI<50% vs LI≥50% | 59.00% | Surgery with preoperative radiation therapy | OS | NA | Univariate and multivariate analysis | 84.40% |
| Ashley H. Davis-Yadley et al. | 2016 | USA | Pancreatic adenocarcinoma | 63 | TNM staging | Low vs high: below the median vs above the median | NA | Surgery | OS | OS: time from surgery to death of patients with pancreatic cancer or last contact, which would be date of last follow-up visit | Multivariate analysis | 85.60% |
| Jeroen A.C.M. Goos et al. | 2015 | Netherlands | Colorectal cancer | 350 | NA | TMA scoring software | NA | Surgery | OS | NA | Multivariate analysis | 86.10% |
| D.G. Hong, et al. | 2012 | Korea | Endometrioid endometrial carcinoma | 41 | FIGO staging system | Negative vs positive: score≤7 vs score>7 | NA | Surgery | OS, DFS | NA | Univariate and multivariate analysis | 81.70% |
| Tineke W.H. Meijer et al. | 2011 | Netherlands | NSCLC | 127 | pTNM 7th edition | Low vs high: staining<13.6% vs ≥13.6% | 50.00% | Surgery | DSS | NA | Kaplan–Meier method | 84.90% |
| Andrej Lyshchik et al. | 2007 | Japan | Pancreatic cancer | 74 | pTNM 6th edition | Low vs high: index<3 vs index≥3 | 44.60% | Surgical treatment and adjuvant chemotherapy | OS | OS: from the date of the surgery to the last follow-up examination | Univariate and multivariate analysis | 83.20% |
| Sara Pizzi et al. | 2009 | Italy | Pancreatic carcinogenesis | 60 | TNM residual tumor classification of the American Joint Committee of Cancer | Low vs high: scores≤50% vs scores>50% | NA | Surgery | OS | NA | Univariate and multivariate analysis | 83.70% |
| Guilherme Cantuaria et al. | 2001 | USA | Ovarian cancinoma | 113 | Stage III-IV | Negative vs positive: staining<50% vs ≥50% | 24.70% | Surgery and adjuvant chemotherapy | DFS | NA | Multivariate analysis | 84.20% |
| Tetsuo Kawamura et al. | 2001 | Japan | Gastric carcinoma | 617 | UICC TNM classification I-IV | Low vs high: scale<30% vs ≥30% | 29.50% | Surgery without chemotherapy and/or irradiation therapy | OS | NA | Univariate and multivariate analysis | 82.50% |
| Sung Soo Kang et al. | 2002 | Korea | Breast carcinoma | 100 | NA | Negative vs positive: staining<50% vs ≥50% | 47.00% | Surgery | OS DFS | NA | Multivariate analysis | 83.30% |
| Richard S. Haber et al. | 1998 | USA | Colorectal carcinoma | 112 | Duke's stage A/B/C/D | Negative vs positive: staining<50% vs ≥50% | 90.00% | Surgery | OS | NA | Multivariate analysis | 83.50% |
| Mamoun Younes et al. | 2001 | USA | Transitional cell carcinoma of the urinary bladder (TCCB) | 40 | AJCC staging system | Low vs high: <10% vs ≥10% | 48.00% | Surgery without adjuvant therapy | OS | NA | Multivariate analysis | 82.70% |
| Hiroyuki Mineta et al. | 2002 | Japan | Hypopharyngeal carcinoma | 99 | 1997 UICC criteria | Low vs high: ≤70% vs >70% | 46.40% | Surgery without any treatment | RFS | NA | Multivariate analysis | 83.20% |
| Youn Wha Kim et al. | 2002 | Korea | Gallbladder carcinomas | 71 | Nevin stage I-V | Low vs high: staining≤50% vs >50% | 52.10% | Surgery | OS | NA | Multivariate analysis | 84.00% |
| Hiroyuki Kato et al. | 2002 | Japan | Esophageal squamous cell carcinoma | 95 | the fifth edition of the TNM classification of the International Union Against Cancer (UICC) | Low vs high: staining≤30% vs >30% | 95.80% | Surgery | OS | NA | Multivariate analysis | 82.80% |

SI(staining index score) or LI(labeling index): staining intensity*proportion of positive stained cells.

OS: overall survival; DFS: disease-free survival; RFS: recurrence-free survival; MFS: metastasis-free survival; CSS: cancer-specific survival; DSS: disease-specific survival.

AJCC: the American Joint Committee on Cancer.

UICC: the International Union Against Cancer.
